# Variability in Care Pathways for Hip Fracture Patients in The Netherlands

**DOI:** 10.3390/jcm13164589

**Published:** 2024-08-06

**Authors:** Hanne-Eva van Bremen, Thamar Kroes, Lotta J. Seppala, Emma A. Gans, Johannes H. Hegeman, Nathalie van der Velde, Hanna C. Willems

**Affiliations:** 1 Amsterdam Bone Center, Movement Sciences Amsterdam, Meibergdreef 9, 1105 AZ Amsterdam, The Netherlands; h.vanbremen@amsterdamumc.nl; 2 Dutch Institute for Clinical Auditing, Rijnsburgerweg 10, 2333 AA Leiden, The Netherlands; 3 Section of Geriatric Medicine, Department of Internal Medicine, Amsterdam UMC, Meibergdreef 9, 1105 AZ Amsterdam, The Netherlands; l.j.seppala@amsterdamumc.nl (L.J.S.); n.vandervelde@amsterdamumc.nl (N.v.d.V.); 4 Amsterdam Public Health Research Institute, Meibergdreef 9, 1105 AZ Amsterdam, The Netherlands; 5 Department of Trauma Surgery, St. Antonius Hospital Utrecht–Nieuwegein, 3543 AZ Utrecht, The Netherlands; t.kroes@antoniusziekenhuis.nl; 6 University Center of Geriatric Medicine, University Medical Center Groningen, Hanzeplein 1, 9713 GZ Groningen, The Netherlands; e.gans@kennisinstituut.nl; 7 Knowledge Institute of the Dutch Association of Medical Specialists, Mercatorlaan 1200, 3528 BL Utrecht, The Netherlands; 8 Biomedical Signals and Systems Group, University of Twente, Drienerlolaan 5, 7522 NB Enschede, The Netherlands; h.hegeman@zgt.nl; 9 Department of Trauma Surgery, Ziekenhuisgroep Twente, Zilvermeeuw 1, 7909 PP Almelo, The Netherlands

**Keywords:** hip fracture, care pathway, integrated hip fracture care

## Abstract

**Background/Objectives**: Integrated orthogeriatric care has demonstrated benefits in hip fracture management for older patients. Comprehensive care pathways are essential for effective integrated care delivery, yet local variability in care pathways persists. We assessed the current hip fracture care pathways in the Netherlands, focusing on the variability between these care pathways and the degree of implementation of orthogeriatric care. **Methods**: A nationwide inventory study was conducted. A survey was sent to all hospitals in the Netherlands to collect the care pathways or local protocols for hip fracture care. All care elements reported in the care pathways and protocols were systematically analyzed by two independent researchers. Furthermore, an assessment was performed to determine which model of orthogeriatric care was applied. **Results**: All 71 Dutch hospitals were contacted, and 56 hospitals responded (79%), of which 46 (82%) provided a care pathway or protocol. Forty-one care elements were identified in total. In the care pathways and protocols, the variability in the description of these individual care elements ranged from 7% to 87%. Twenty-one hospitals had an integrated care model with shared responsibility, while an equal number followed an orthopedic trauma surgeon-led care model. **Conclusions**: These findings provide a detailed description of the hip fracture care pathways in the Netherlands. Variations were observed concerning the care elements described in the care pathways, the structure of the care pathway, and the specification of several elements. The implementation of integrated care with shared responsibilities, as recommended by the international literature, has not been achieved nationwide. The clinical implications of the variability between care pathways, such as the influence on the quality of care, need to be further investigated.

## 1. Introduction

Integrated orthogeriatric care has significant benefits for older patients with a hip fracture, including improved quality of life, reduced morbidity and mortality, increased activities of daily living at 6 and 12 months, and reduced direct costs in comparison to usual care [1,2]. Hip fracture patients are distinct due to the high prevalence of multimorbidity and increased frailty [3]. These patient characteristics are associated with prolonged hospital stays, delays in surgery, a functional decline, and increased mortality rates [4]. Given these patient characteristics and risk factors, the treatment of hip fracture patients is complex and requires the integration of both surgical and geriatric care to adequately address comorbidities and geriatric syndromes [1].

In the literature, different models of integration in orthogeriatric trauma care are described: (1) orthopedic trauma surgeon-led care with a geriatrician as a consulting physician; (2) geriatrician-led care with consultation from an orthopedic trauma surgeon, and (3) integrated care models with shared responsibilities between a geriatrician and orthopedic trauma surgeon [5,6,7]. In the integrated care model, orthogeriatric care can be implemented as an integral component of the orthogeriatric ward. This has demonstrated positive effects on both patient outcomes and the quality of care, including reduced lengths of stay in the hospital and emergency department and shorter times to operation [8,9,10].

The integration of surgical and geriatric care for hip fracture patients is preferably coordinated in care pathways that address both clinical and organizational issues. In the current literature, several synonyms exist for the term ‘care pathway’, such as clinical pathway, model of care, and integrated care pathway. It is defined as an integrated care plan that outlines patient goals and provides the sequence and timing of the necessary actions to achieve these goals [11,12]. Several care pathways are known for a various number of conditions. The use of care pathways enhances the efficiency and quality of care for hip fracture patients. It can also be used as a method to implement evidence-based clinical guidelines in practice [13].

Despite the added value of care integration in orthogeriatric hip fracture care, the implementation of orthogeriatric care remains inconsistent across hospitals in the Netherlands and between countries in Europe [14,15]. Although a certain level of local variability in care processes across hospitals is inevitable, comprehensive protocols are important for integrated care delivery [16,17,18]. Therefore, the aim of this study was to assess the current hip fracture care pathways and protocols in the Netherlands for variability between the pathways by evaluating the different elements and the degree of implementation of orthogeriatric care.

## 2. Materials and Methods

A cross-sectional national inventory study was conducted from 1 May 2023 until 1 October 2023. All hospitals across the Netherlands were asked to provide their protocols regarding hip fracture care. If the hospitals did not respond to the initial request sent through professional organizations, they were contacted directly via telephone and email for their care pathway protocol or protocols for hip fracture patients. Recognizing the multidisciplinary care approach, both the geriatric and surgical departments were asked to provide the documents. All protocols that specifically addressed the treatment of hip fracture patients were included. Additional information per email and personal experiences shared by healthcare professionals were excluded from the analysis as the scope of this study focused on the assessment of local authorized protocols and care pathways.

### 2.1. Data Analysis

#### 2.1.1. Screening Tool

No validated method was available for the evaluation of local care pathways. Previous research into systematic reviews assessing the quality of care pathways did not establish a gold standard for screening [19]. When asked for a recommendation by researchers in the field, the European Pathway Association (EPA) stated that there are currently no suitable tools for the comparison of local (hip fracture) protocols or care pathways.

In the absence of a validated method, a manifest content analysis was conducted on all retrieved protocols [20]. This entailed (1) the identification of the care elements within the care pathways and (2) examining what was documented about the care elements across the care pathways. Within several care elements, variability was observed and further explored, encompassing the type of diagnostics (emergency care), treatment team, use of thrombosis prophylaxis, involvement of paramedics (pre- and post-operative), and organization of outpatient visits.

#### 2.1.2. Content Analysis

The content analysis was conducted using alternate deductive and inductive methods. Efforts were made to ensure the robustness of the approach. Before the analysis, a content model of care elements was established based on the national Dutch hip fracture care guidelines [21]. This content model was modified and approved by an expert panel including geriatricians, internists, orthopedic surgeons, trauma surgeons, and physiotherapists. After the content model was established, two researchers (H.B. and T.K.) independently screened all protocols and noted the presence of care elements in a deductive manner. Furthermore, the researchers used this initial screening to reveal the content of the care elements and identify additional care elements in an inductive manner. After intercoder agreement was achieved (H.B., T.K., H.W.), several care elements were added to the content model. A second screening was conducted to note both the inclusion and content of the newly identified care elements in all protocols (H.B., T.K.). After the second round, the independent evaluations were assessed for differences, and inter-coder agreement was reached (H.B., T.K.). In instances of non-agreement, a third researcher (H.W.) was available to resolve discrepancies.

The intention was to categorize the care elements according to Donabedian’s healthcare quality model to identify the protocols’ structural, process, and outcome elements [22]. However, all identified care elements were process-related. Consequently, the researchers chose to report the care elements by following the care pathway of the Orthogeriatric Care Model of the FFN, encompassing the emergency phase, pre-operative phase, operative phase, post-operative phase, rehabilitation, and return to function [23]. A non-operative phase was added separately since it recurred in several care pathways. Structural elements were identified within the care pathways: in- and exclusion criteria, the use of quality indicators, and responsibility tables or flowcharts.

#### 2.1.3. Model of Orthogeriatric Care

The type of integrated care in orthogeriatric trauma was divided into three different types: (1) orthopedic trauma surgeon-led care—a surgeon-led model with the patient on an orthopedic/surgical ward and consultation with the geriatrician; (2) geriatrician-led care—a geriatrician-led model with the patient on a geriatric ward and consultation with an orthopedic trauma surgeon; (3) an integrated care model with shared responsibility—the patient on a ward with shared care between a geriatrician and orthopedic trauma surgeon [5,6,7]. The model used in the hospital/care pathway was identified and independently described by the two researchers (H.B., T.K.). In case of disagreement, a third researcher (H.W.) was available.

## 3. Results

### 3.1. Inventory

**Figure 1:**
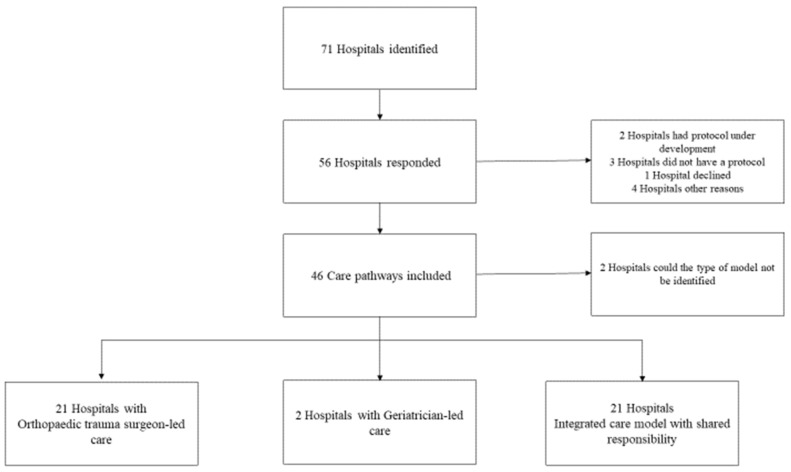
Flowchart with the inventory process and distribution of orthogeriatric care models.

Out of 71 contacted hospitals, 56 responded (response rate 79%). Among them, 46 (82%) hospitals shared their care pathways. Four of the responding hospitals were affiliated with universities. Ten hospitals could not provide a care pathway: two had a care pathway under development, three did not have one, four hospitals did not share a written protocol but instead provided information via telephone and details about the organization, and one declined to provide it. For 44 hospitals, the type of orthogeriatric care model could be identified. The distribution was equal between hospitals with orthopedic trauma surgeon-led care (*N* = 21) and those with an integrated care model with shared responsibilities (*N* = 21). Two hospitals had geriatrician-led care (Figure 1).

### 3.2. Identified Care Elements

**Figure 2:**
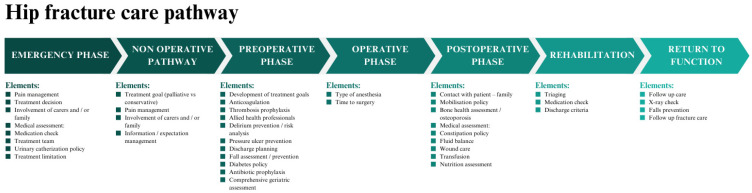
Overview of identified care elements per phase of care.

**Figure 3:**
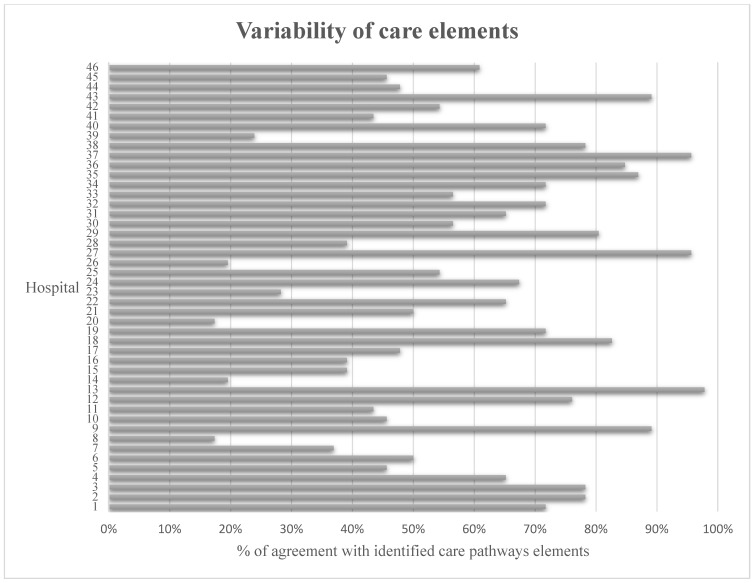
Percentage of agreement with the identified care elements per hospital.

A detailed overview of the care elements with a description of the content is provided in Figure 2. In total, 43 care elements were identified by the content analysis. The presence of the care elements varied between 7% (information–expectation management in the non-operative phase) and 87% (delirium prevention/risk analysis) (Figure 3). Several elements were present in >70% of the hip fracture care pathways, including pain management, medical assessment—blood tests and electrocardiograms, a urinary catheterization policy, thrombosis prophylaxis, delirium prevention/risk analysis, discharge planning, and a mobilization policy (Table 1). Various care elements were present in less than 20% of the hip fracture care pathways, including pain management in the non-operative phase, the involvement of carers and/or family, and information/expectation management in the non-operative phase.

### 3.3. Structure of Care Pathway

The care pathways varied in their inclusion and exclusion criteria and the use of quality indicators. Several care pathways applied an age threshold for patients ≥ 70 years old (*N* = 7) or only included frail patients (*N* = 4), and four care pathways excluded pathological hip fractures.

Out of 46 care pathways, 19 described quality indicators. An overview of the identified quality indicators can be found in Table 2. The quality indicator “Operation within 24 h after admission” was most often described (*N* = 9). Flowcharts and responsibility formats were identified as structural elements. Specifically, 11 included a flowchart of the care pathway, and eight included a responsibility matrix specifying which healthcare provider was responsible for each part of the care.

### 3.4. Care Elements

#### 3.4.1. Diagnostics (Emergency Care)

Among the 46 care pathways, 39 described information regarding the medical assessment conducted in the Emergency Department. Various types of diagnostics were identified, including chest X-rays, electrocardiograms, blood tests, and urine sediment analyses. Specifically, 38 hospitals recommended blood tests, 35 mentioned electrocardiograms, 27 described chest X-rays, and 17 specified urine sediment analyses.

#### 3.4.2. Treatment Team

The attending physician was, in most cases, the orthopedic surgeon or a trauma-certified surgeon (*N* = 16), and the geriatrician was the consulting physician (*N* = 15). In limited cases, the geriatrician acted as an attending physician (*N* = 2). In some care pathways, a change in the treatment team occurred during the hospital stay, where the geriatrician took over the role of the attending physician from the surgeon after 24 h (*N* = 1), directly post-operatively (*N* = 2), or after two days (*N* = 3).

#### 3.4.3. Use of Thrombosis Prophylaxis

Different types and durations of thrombosis prophylaxis post-operatively were observed in 35 care pathways. In most cases, a separate care protocol was described for the use of thrombosis prophylaxis (*N* = 8). Dalteparin was mentioned most often (*N* = 8), with durations varying from 5 weeks (*N* = 4) to 6 weeks (*N* = 1). Seven care pathways advised the use of Fraxiparine for 4 to 6 weeks, and two recommended Nadroparin.

#### 3.4.4. Involvement of Allied Health Professionals (Pre- and Post-Operative)

Out of the 46 care pathways, 33 described the consultation of paramedics either pre- or post-operatively. Four types of paramedics were identified: physiotherapy, dietetics, speech therapy, and occupational therapy. Regarding physiotherapy, four care pathways recommended a pre-operative consultation, 13 care pathways a post-operative consultation, and 24 did not specify the timing of consultation. A dietetics consultation was recommended, in most cases, in the case of malnutrition (*N* = 16). Six care pathways described the consultation of a speech therapist, and one care pathway described the consultation of an occupational therapist.

#### 3.4.5. Outpatient Visits

Among the 46 care pathways, the timing of the outpatient visits could be identified in 34 pathways. Most recommended a follow-up visit six weeks post-operatively (*N* = 24), and 19 care pathways advised a follow-up visit at three months. Other care pathways advised to plan a visit at 8–10 weeks (*N* = 1), six months (*N* = 2), and 12 months (*N* = 2). Three care pathways recommended to forego outpatient follow-up in the hospital in case the patient was discharged to a nursing home. Two care pathways recommended to forego follow-up in case of cognitive impairment or dementia.

## 4. Discussion

A descriptive content analysis was conducted to map the orthogeriatric care integration and variability within care pathways in the Netherlands. Forty-three care elements were identified in 46 different care pathways, with substantial variability in the care pathway elements, ranging from 7% to 87%. Further analysis uncovered variability regarding the treatment team, the involvement of allied health professionals, thrombosis management, and post-operative care. The models of orthogeriatric care were divided equally between orthopedic trauma surgeon-led care with geriatric consultation (*N* = 21) and integrated care with shared responsibilities (*N* = 21).

Standardizing clinical processes is an effective strategy in reducing variability and minimizing the risk of medical errors [24]. In this study, substantial variability was observed in several aspects of the care pathways. The variation in care elements as well as treatment policies might therefore be concerning. For example, certain elements, such as delirium prevention, urinary catheterization policies, and discharge planning, occurred relatively frequently, while osteoporosis care was seldom described. Hip fracture patients constitute a heterogeneous group where a uniform approach to care may not always be appropriate. However, it can be argued that certain elements warrant almost universal application, such as having integrated or shared decision-making and a pain management plan, as advocated for by the FFN. Several of these elements, where universal application could be argued, were infrequently described in the care pathways, including the development of treatment goals. Although some space for variability in the clinical application of care is necessary due to the heterogeneity of the hip fracture population, the further standardization of hip fracture care pathways might be a promising improvement strategy.

A prerequisite for the further standardization of hip fracture care pathways is a clear definition of the term ‘care pathway’. This is necessary to provide direction for clinicians and establish the grounds for the adequate evaluation of care pathways. In the current literature, several synonyms and definitions exist for the term ‘care pathway’, and clear descriptions of the care pathway evaluation criteria are lacking. The broad terminology used for the term care pathway also complicates the process of identifying the relevant literature and evaluation methods. To our knowledge, a validated method to evaluate care pathways remains absent. Most literature describes single-site implementations and the implementation of care pathways. It does not examine multiple care pathways on a broad scale, as was conducted in the current study, which looked at the national level. A previous systematic review by Vanhaecht identified various tools to assess care pathways [25]. This review identified the Integrated Care Pathways Appraisal Tool (ICPAT) as the most appropriate tool to evaluate clinical pathway documents; however, it was neither available nor used in practice and mainly focused on the English care setting rather than the Dutch [26,27]. Vanhaecht et al. concluded that extremely little research has been conducted on these tools. The available tools often have a specific application for the implementation and evaluation of the care pathway, work in a particular setting, or are not validated or widely used. Since no validated method was available, the deductive and inductive content analysis made it possible to evaluate all available care pathways critically and understand the variability between the care elements. Therefore, all efforts were made to sustain a robust and valid method for the assessment of the care pathways through evaluation by two independent researchers and contacting the European Pathway Association (EPA) for alternatives.

Besides the ambiguous definition of the term care pathway and the subsequent complicated evaluation, there remains a gap in understanding how care pathways and protocols translate into everyday clinical practice. Consequently, it is unknown how differences within care pathways and protocols between hospitals influence the quality of care. However, the implementation of a care pathway for older patients with hip fractures has been shown to improve the quality of care and is advised by the FFN [28]. As previously described, reducing variability through the standardization of clinical processes, of which a care pathway is one, effectively reduces the risk of medical errors [24]. However, it is important to consider the lessons from the earlier literature that discusses the balance between clinicians’ freedom and the autonomy to provide appropriate care [29]. In other words, care pathway implementation does not equal ‘cookbook medicine’ and this balance is important for successful care pathway implementation.

In this study, almost half of the hospitals in the Netherlands used the integrated care model with shared responsibility. Previous studies favored the integrated care model with shared responsibility and geriatrician-led care over orthopedic trauma surgeon-led care in terms of better outcomes [5,6,7]. Although the evidence for one model over the other is limited, the overall evidence for orthogeriatric or multidisciplinary care over usual care is apparent. Solberg et al. showed better outcomes for integrated care in comparison to orthopedic trauma surgeon-led care regarding the removal of a urinary catheter, mobilization day one post-operatively, and secondary fracture prevention [30]. In a literature review, Kammerlander et al. compared four different models and found favorable outcomes for integrated care regarding in-hospital mortality, the length of stay, and the mean time to surgery [31]. Van Heghe et al., in a systematic review and meta-analysis, compared the three models used in our study and observed better outcomes for all models in terms of the length of stay, in-hospital mortality, 1-year mortality, and delirium [7]. However, they could not conclusively recommend one orthogeriatric care model over another. Schuijt et al. evaluated the outcomes before and after implementing an integrated orthogeriatric trauma unit and showed a reduction in post-operative complications, lower 1-year mortality, less time spent at the ED, and better data registration [9]. A previous study by Werner et al. found the lowest percentage of joint care in the Netherlands at 74%, compared to the data of other European hip fracture registrations [14]. Since the evidence regarding the benefits of integrated care has been known for several years, this raises the question of why integrated care is not applied in most protocols. The analyzed protocols may provide an incomplete picture of the actual clinical practice due to outdated or incomplete care pathways. Although a previous study found support for integrated care among specialists, including geriatricians and orthopedic trauma surgeons, there may be unknown barriers in the Dutch healthcare system that delay the implementation of integrated care, such as financial reasons or a lack of availability of physicians [32].

The strengths and limitations of this study should be considered. We conducted extensive research looking into local written care pathways in the hospitals, seeking to bridge the gap between the care pathway literature and the real-world hospital setting and explore the gap between the care pathway literature and their implementation in hospitals. The method used in this study made it possible to perform an extensive analysis of the care pathways and elucidate the existing variability. The limitations included the lack of a validated care pathway evaluation method, which resulted in the authors performing a descriptive content analysis. Secondly, we merely explored the documented care pathways and did not evaluate clinical practice. While it is likely that variability in protocols results in variability in clinical practice, this cannot be concluded with certainty.

## 5. Conclusions

These findings show substantial variability in hip fracture care pathways in the Netherlands. Variations were observed concerning the frequency of care elements described in the care pathways, ranging from 7% to 87%. Additionally, the structure and specifications of specific elements, such as thrombosis prophylaxis and outpatient visits, varied widely. As integrated care with shared responsibilities was observed in less than half of the hospitals, it seems to lag behind in relation to the current scientific recommendations. The clinical implications of the variability between care pathways, such as its influence on the quality of care, need to be further investigated. Future research should focus on developing clear care pathway definitions and evaluation methods.

## Figures and Tables

**Table 1:** Overview of identified care elements.

Phase	Elements of Care Pathways	Total *N* = 46 *N* (%)
Emergency phase	Pain management	35 (76)
	Treatment decision	28 (61)
	Involvement of carers and/or family	18 (39)
	Medical assessment: chest X-ray urine analysis blood test electrocardiogram	27 (59) 17 (37) 38 (83) 35 (76)
	Medication check	24 (52)
	Treatment team attending physician consulting physician	17 (37) 12 (26)
	Urinary catherization policy	33 (72)
	Treatment limitation	29 (63)
Non-operative pathway	Treatment goal (palliative vs. conservative)	17 (37)
	Pain management	7 (15)
	Involvement of carers and/or family	5 (11)
	Information/expectation management	3 (7)
Pre-operative phase	Development of treatment goals	20 (43)
	Anticoagulation	25 (54)
	Thrombosis prophylaxis	36 (78)
	Allied health professionals physiotherapist dietician	29 (63) 20 (43)
	Delirium prevention/risk analysis	40 (87)
	Pressure ulcer prevention	27 (59)
	Discharge planning	37 (80)
	Fall assessment/prevention	30 (65)
	Diabetes policy	12 (26)
	Antibiotic prophylaxis	19 (41)
	Comprehensive geriatric assessment	10 (22)
Operative phase	Type of anesthesia	10 (22)
	Time to surgery	18 (39)
Post-operative phase	Contact with patient/family	25 (54)
	Mobilization policy	33 (72)
	Bone health assessment/osteoporosis	25 (54)
	Medical assessment: X-ray blood test	22 (48) 19 (41)
	Constipation policy	18 (39)
	Fluid balance	26 (57)
	Wound care	26 (57)
	Transfusion	16 (35)
	Nutrition assessment	27 (59)
Rehabilitation	Triaging	31 (67)
	Medication check	22 (48)
	Discharge criteria	10 (22)
Return to function	Follow-up care two weeks six weeks three months	13 (28) 26 (57) 19 (41)
	X-ray check	21 (46)
	Fall prevention	16 (35)
	Follow-up fracture care	28 (61)

**Table 2:** Identified quality indicators used in the care pathways.

	Quality Indicators	*N*
Process	Discharge from the emergency department to a ward within a 1 h timeframe 90 min timeframe 2 h timeframe	2 1 6
	Assessed by a doctor within 2 h	1
	Operation within X hours after admission 24 h Within 24 h in case of ASA 1-2 36 h Median time between operation–admission	9 1 1 1
	Discharge from hospital Length of stay Discharge on day 4–5 % Delayed length of stay (>day 4) 90% Within length of stay of 6 days	1 1 1 1
	Co-treatment by a geriatrician Patients operated by a certified trauma surgeon or orthopedic surgeon and peri-operative co-management by a geriatrician Co-management in patients over 70 years old 100% assessment by a geriatrician within 1 day of admission	1 1 1
	Registry Registration of functional outcome measurement three months after discharge Registration of functional outcome measurements for patients over 70 years old Registration of functional measurements before admission and three months after discharge	1 1 1
	Percentage of patients with an FICB	
Outcome	Reoperation Within three months due to wound infection Within 60 days in patients over 65 years old	1 1
	Informed consent check 100%	1
	Complications (wound infection, pressure ulcer, delirium)	1
	Mortality after 30 days and after one year	1
	Pain score NRS 4	1
	Readmissions (not further specified)	1
	Surgery report within 24 h	1
	Discharge letter within 24 h	1
	Morbidity (not further specified)	1
	Patients aged 50–80 and over 80 with a bone mineral density (BMD) measurement within 1 year before or up to 3 months after a fracture	1

## Data Availability

The data presented in this study are available on request from the corresponding author and hospital.

## References

[1] Baroni M., Serra R., Boccardi V., Ercolani S., Zengarini E., Casucci P., Valecchi R., Rinonapoli G., Caraffa A., Mecocci P. (2019). The Orthogeriatric Comanagement Improves Clinical Outcomes of Hip Fracture in Older Adults. Osteoporos. Int..

[2] Hsu Y.F., Chou F.H., Wang H.H., Chu Y.C., Liao K.L. (2023). Effectiveness of Integrated Care for Elderly Patients with Hip Fractures: A Systematic Review and Meta-Analysis. Geriatr. Nurs..

[3] Baker P.N., Salar O., Ollivere B.J., Forward D.P., Weerasuriya N., Moppett I.K., Moran C.G. (2014). Evolution of the Hip Fracture Population: Time to Consider the Future? A Retrospective Observational Analysis. BMJ Open.

[4] Roche J.J.W., Wenn R.T., Sahota O., Moran C.G. (2005). Effect of Comorbidities and Postoperative Complications on Mortality after Hip Fracture in Elderly People: Prospective Observational Cohort Study. Br. Med. J..

[5] Grigoryan K.V., Javedan H., Rudolph J.L. (2014). Orthogeriatric Care Models and Outcomes in Hip Fracture Patients: A Systematic Review and Meta-Analysis. J. Orthop. Trauma.

[6] Patel J.N., Klein D.S., Sreekumar S., Liporace F.A., Yoon R.S. (2020). Outcomes in Multidisciplinary Team-Based Approach in Geriatric Hip Fracture Care: A Systematic Review. J. Am. Acad. Orthop. Surg..

[7] Van Heghe A., Mordant G., Dupont J., Dejaeger M., Laurent M.R., Gielen E. (2022). Effects of Orthogeriatric Care Models on Outcomes of Hip Fracture Patients: A Systematic Review and Meta-Analysis. Calcif. Tissue Int..

[8] Mangram A.J., Shifflette V.K., Mitchell C.D., Johnson V.A., Lorenzo M., Truitt M.S., Goel A., Lyons M., Dunn E.L. (2011). The Creation of a Geriatric Trauma Unit “G-60”. Am. Surg..

[9] Schuijt H.J., Kusen J., van Hernen J.J., van der Vet P., Geraghty O., Smeeing D.P.J., van der Velde D. (2020). Orthogeriatric Trauma Unit Improves Patient Outcomes in Geriatric Hip Fracture Patients. Geriatr. Orthop. Surg. Rehabil..

[10] Flikweert E.R., Wendt K.W., Diercks R.L., Izaks G.J., Stewart R., Stevens M., Reininga I.H.F. (2021). A Comprehensive Multidisciplinary Care Pathway for Hip Fractures Better Outcome than Usual Care?. Injury.

[11] Campbell H., Hotchkiss R., Bradshaw N., Porteous M. (1998). Integrated Care Pathways. BMJ.

[12] Every N.R., Hochman J., Becker R., Kopecky S., Cannon C.P. (2000). Critical Pathways: A Review. Committee on Acute Cardiac Care, Council on Clinical Cardiology, American Heart Association. Circulation.

[13] Rotter T., Kinsman L., James E.L., Machotta A., Gothe H., Willis J., Snow P., Kugler J. (2010). Clinical Pathways: Effects on Professional Practice, Patient Outcomes, Length of Stay and Hospital Costs. Cochrane Database Syst. Rev..

[14] Werner M., Macke C., Gogol M., Krettek C., Liodakis E. (2022). Differences in Hip Fracture Care in Europe: A Systematic Review of Recent Annual Reports of Hip Fracture Registries. Eur. J. Trauma Emerg. Surg..

[15] Würdemann F.S., Krijnen P., van Zwet E.W., Arends A.J., Heetveld M.J., Trappenburg M.C., Hegeman J.H., Schipper I.B., Calf A.H., van Egmond P.W. (2022). Trends in Data Quality and Quality Indicators 5 Years after Implementation of the Dutch Hip Fracture Audit. Eur. J. Trauma Emerg. Surg..

[16] Fox F., Drew S., Gregson C.L., Patel R., Chesser T.J.S., Johansen A., Javaid M.K., Griffin X.L., Gooberman-Hill R. (2023). Complex Organisational Factors Influence Multidisciplinary Care for Patients with Hip Fractures: A Qualitative Study of Barriers and Facilitators to Service Delivery. BMC Musculoskelet. Disord..

[17] Suter E., Oelke N.D., Adair C.E., Armitage G.D. (2009). Ten Key Principles for Successful Health Systems Integration. Healthc. Q..

[18] Evans-Lacko S., Jarrett M., McCrone P., Thornicroft G. (2010). Facilitators and Barriers to Implementing Clinical Care Pathways. BMC Health Serv. Res..

[19] Latina R., Salomone K., D’angelo D., Coclite D., Castellini G., Gianola S., Fauci A., Napoletano A., Iacorossi L., Iannone P. (2020). Towards a New System for the Assessment of the Quality in Care Pathways: An Overview of Systematic Reviews. Int. J. Environ. Res. Public Health.

[20] Kleinheksel A.J., Rockich-Winston N., Tawfik H., Wyatt T.R. (2020). Demystifying Content Analysis. Am. J. Pharm. Educ..

[21] Startpagina—Proximale Femurfracturen-Richtlijn-Richtlijnendatabase. https://richtlijnendatabase.nl/richtlijn/proximale_femurfracturen/proximale_femurfracturen_-_startpagina.html.

[22] Moore L., Lavoie A., Bourgeois G., Lapointe J. (2015). Donabedian’s Structure-Process-Outcome Quality of Care Model: Validation in an Integrated Trauma System. J. Trauma Acute Care Surg..

[23] Fragility Fracture Network Orthogeriatric Care Model. https://fragilityfracturenetwork.org/wp-content/uploads/2023/10/ocm-2-pdf.pdf.

[24] Kohn L.T., Corrigan J.M., Donaldson M.S. (2000). To Err Is Human: Building a Safer Health System.

[25] Vanhaecht K., De Witte K., Depreitere R., Sermeus W. (2006). Clinical Pathway Audit Tools: A Systematic Review. J. Nurs. Manag..

[26] Whittle C. (2009). ICPAT: Integrated Care Pathways Appraisal Tool. Int. J. Care Pathways.

[27] Whittle C.L., Mcdonald P.S., Dunn L., de Luc K. (2004). Developing the Integrated Care Pathway Appraisal Tool (ICPAT): A Pilot Study. Int. J. Care Pathways.

[28] Falaschi P., Marsh D. (2021). Orthogeriatrics: The Management of Older Patients with Fragility Fractures.

[29] Panella M., Marchisio S., Di Stanislao F. (2003). Reducing Clinical Variations with Clinical Pathways: Do Pathways Work?. Int. J. Qual. Health Care.

[30] Solberg L.B., Vesterhus E.B., Hestnes I., Ahmed M.V., Ommundsen N., Westberg M., Frihagen F. (2023). Comparing Two Different Orthogeriatric Models of Care for Hip Fracture Patients: An Observational Prospective Cross-Sectional Study. BMJ Open Qual..

[31] Kammerlander C., Roth T., Friedman S.M., Suhm N., Luger T.J., Kammerlander-Knauer U., Krappinger D., Blauth M. (2010). Ortho-Geriatric Service-a Literature Review Comparing Different Models. Osteoporos. Int..

[32] van Bremen H.E., Seppala L.J., Gans E., Johannes H., van der Velde N., Willems H.C. (2024). Defining Optimal Orthogeriatric Hip Fracture Care: A Delphi Consensus Approach.

